# Editorial: Emerging pathogens and contaminants in water matrices: human health risks, exposure pathways and epidemiological outcomes

**DOI:** 10.3389/fmicb.2023.1283192

**Published:** 2023-09-20

**Authors:** Anthony A. Adegoke, Olayinka A. Aiyegoro, Sheena Kumari

**Affiliations:** ^1^Department of Microbiology, Faculty of Science, University of Uyo, Uyo, Nigeria; ^2^Department of Community Health Studies, Faculty of Health Sciences, Durban University of Technology, Durban, KwaZulu-Natal, South Africa; ^3^Research Unit for Environmental Sciences and Management, North West University, Potchefstroom, North West, South Africa; ^4^Institute for Water and Wastewater Technology, Durban University of Technology, Durban, KwaZulu-Natal, South Africa

**Keywords:** emerging pathogens, emerging chemicals, exposure pathways, wastewater reuse, contaminated food cycle, human health risks, source tracking and mitigation steps

## 1. Conceptualization

The research topic was conceived during a Nigerian-based research project sponsored by the National Research Fund (NRF) of the TETFUND, Project Code TETF/DR&D-CE/NRF/2020/SETI/99/VOL.1. The objective of this research was to address the quantitative risk assessment of *Listeria monocytogenes* in food grown using organic fertilizers. Wastewater, an integral component of water matrices, is employed for irrigating food crops, being nutrient as a source of organic fertilizers. However, it is important to note that wastewater-irrigated and other organically grown food crops can pose risks to human health if appropriate pretreatment and decontamination measures are not followed.

## 2. Emerging pathogens and human health risks

Emerging pathogens exist within the realm of microorganisms, within which exist more potent pathogens. Their presence within water matrices signifies a significant epidemiological threat, especially concerning crops irrigated with such water. This potential hazard can also directly impact humans who come into contact with these pathogens, either through accidental ingestion in recreational water or inhalation of aerosols generated from such water sources (Adegoke et al., 2018).

**Figure 1:**
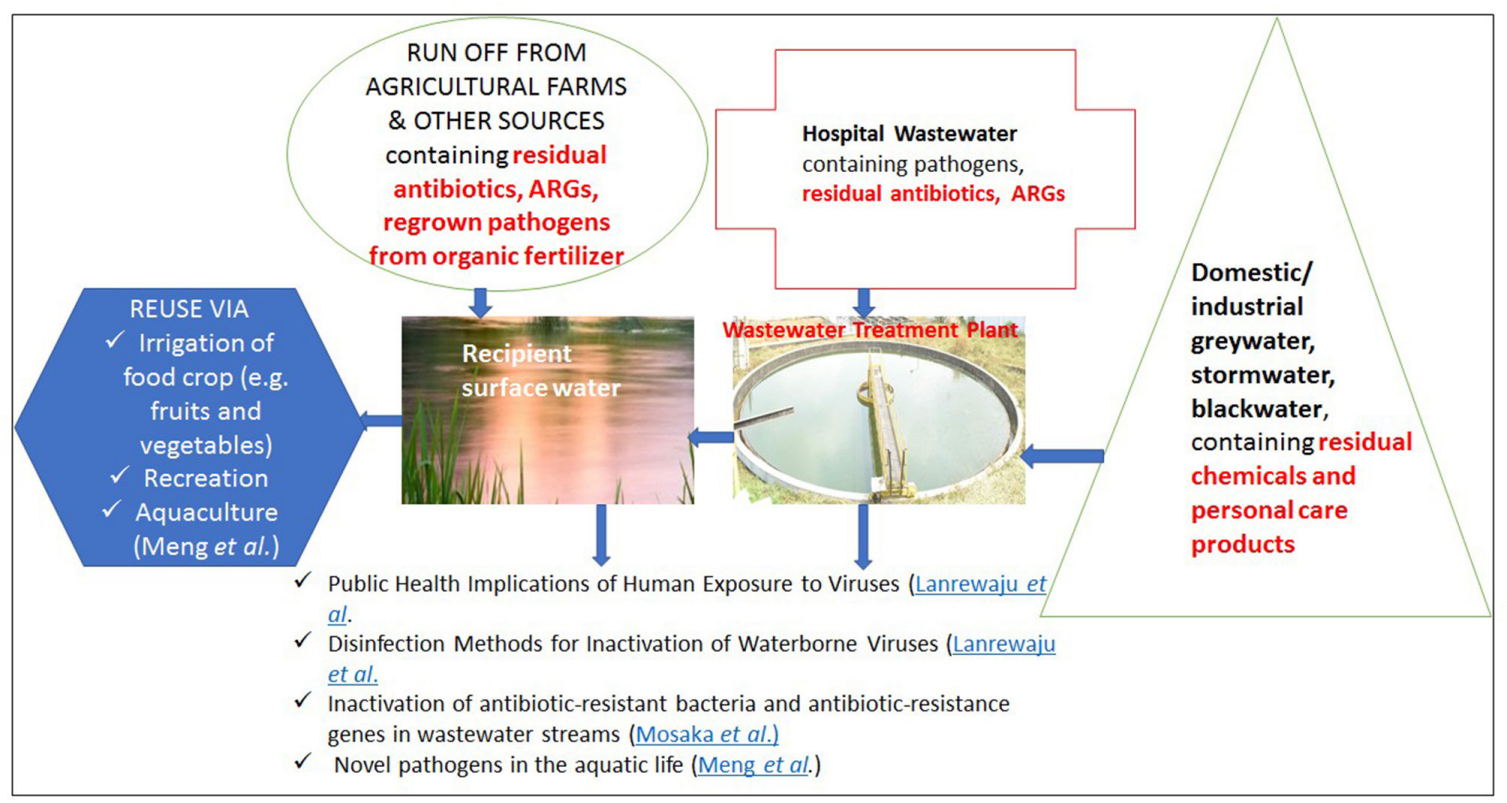
Few illustrated pathways for recycling pathogens and contaminants in and out of water matrices.

Pathogens in water ecosystems pose a threat not only to consumers of ready-to-eat crops but also to the fauna, whether they are being raised domestically or are part of the wild fauna inhabiting such contaminated water habitats. Within the scope of this Research Topic, Figure 1 illustrates several pathways through which pathogens can circulate within water matrices.

Lanrewaju, Enitan-Folami, Sabiu, Edokpayi et al. conducted a review focusing on enteric viruses like adenovirus, rotavirus, noroviruses, and enteroviruses including coxsackievirus, poliovirus, etc. These viruses have been linked to gastroenteritis and severe conditions like encephalitis, hepatitis, cancer, and myocarditis. They stressed the need to monitor and remove enteric viruses from water sources, highlighting public health concerns and the importance of regular viral monitoring in treated wastewater before release into the environment.

Efficiencies of wastewater-based epidemiology (WBE) have been proven to align with the saliva-based methods by Ash et al. in determining the presence of SARS-CoV-2, using the CDC primers for N Capsid targets N1 and N3 via the RT-qPCR. This further affirms that wastewater that usually harbors these pathogens can serve as early warning sign of the health of the population.

Lanrewaju, Enitan-Folami, Sabiu, Swalaha reviewed prevalent viral disinfection methods for wastewater. They assessed various techniques, considering their pros, cons, and log reduction values for virus removal. These methods include physical (e.g., membrane filtration, UV irradiation) and chemical (e.g., chlorine, chlorine dioxide) approaches. The study also explored emerging technologies like photocatalysis, cavitation, and electrochemistry, comparing them to conventional methods. Additionally, it examined health risks from disinfection by-products, such as bladder cancer and reproductive issues.

The pathogens of concern are diverse and include bacterial pathogens. In their study, Meng et al. identified a new pathogenic form of *Kocuria kristinae* found in *Larimichthys crocea*, a marine fish species. They observed significant genomic variations among *K. kristinae* strains, possibly reflecting adaptations to different environments. A regression test using *L. crocea* demonstrated the bacterium's pathogenicity, causing dose-dependent mortality within 5 days post-infection, affirming its threat to marine fish.

Since accuracy and promptness are vital in the detection of bacterial pathogens from the water or wastewater for reuse, the methods employed should guarantee these factors. Yang et al. carried out a study to rapidly and accurately detect Toxigenic *Vibrio cholerae* serogroups O1 and O139 in environmental water. The researchers combined triplex droplet digital PCR (ddPCR) with propidium monoazide (PMA) treatment to improve traditional plate counting, which is time-consuming. The method utilized specific primers and probes, optimized conditions, and demonstrated superior sensitivity and specificity compared to qPCR. PMA-ddPCR offers a faster and more precise means of detecting these cholera-causing pathogens in suspicious seawater samples.

## 3. Emerging contaminants and human health risks

Residual antibiotics, antibiotic resistant bacteria (ARBs) and antibiotic resistance genes (ARGs) among others remain in the category of emerging pathogens. These follow similar pathways as illustrated in Figure 1. Their impacts include, but not limited to the threat of limiting treatment options, high cost of treatment and prolong hospital stay, when a susceptible individual gets infected by the ARBs bearing ARGs. Mosaka et al. highlighted WWTPs, antibiotic usage, ARBs, and ARGs' interconnectedness crucial for understanding AMR epidemiology. Traditional WWTPs' limitations in handling antibiotics led to evaluating cold atmospheric plasma (CAP) as an eco-friendly alternative. CAP disinfects wastewater, augments bioelectricity, making CAP integration in future WWTPs advisable.

## 4. Effects on crop, exposure pathways, and mitigation strategies

Crops such as lettuce, spinach, carrots, garden egg, cabbage, and cucumbers worldwide face contamination by emerging viral, bacterial, and parasitic pathogens and contaminants due to irrigation with wastewater or organic fertilizers (Finley et al., 2013; Zhang et al., 2015; Adegoke et al., 2016). These pathogens can infiltrate and internalize within crops, making surface washing ineffective, thus increasing health risks for consumers. Farmers and workers handling these crops also encounter pathogens, impacting consumers through groundwater contamination. Sprinkler irrigation, commonly used in farming, can further expose communities to diseases linked to wastewater reuse (Adegoke et al., 2018). Consuming produce from such farms introduces additional risks, necessitating the urgent management of these complex health threats.

Disinfecting wastewater and surface water for crop irrigation and other reuse is crucial, requiring prompt, reliable methods (Lanrewaju, Enitan-Folami, Sabiu, Swalaha). Quality assessments of the water for reuse, emphasizing on methods with high sensitivity and specificity, are also recommended before reuse (Yang et al.).

## Author contributions

AA: Conceptualization, Writing—original draft, Writing—review and editing. OA: Validation, Writing—review and editing. SK: Validation, Writing—review and editing.
